# YOLO-AMI: enhancing online quality monitoring in 3D printing with composite loss and parameter-free attention

**DOI:** 10.1038/s41598-026-44342-6

**Published:** 2026-04-11

**Authors:** Zhaoxuan Li, Mohd Salman Abu Mansor

**Affiliations:** School of Mechanical Engineering, Tuanku Syed Sirajuddin Engineering Campus, Universiti Sains Malaysia, Seri Ampangan, 14300 Nibong Tebal, Seberang Perai Selatan, Pulau Pinang Malaysia

**Keywords:** Additive manufacturing, Deep learning, Small object detection, YOLOv10, Engineering, Mathematics and computing

## Abstract

Additive manufacturing (AM) is transforming industrial production; however, inevitable defects—such as spaghetti-like collapses, surface blemishes (“zits”), and stringing—substantially degrade product quality and mechanical performance. To overcome limitations of traditional inspection methods—often inefficient, subjective, or reliant on costly offline equipment—a high-precision, real-time defect-detection model, YOLO-AMI, is proposed, based on the YOLOv10 architecture. The neck network was reconstructed using the Asymptotic Feature Pyramid Network to enhance multi-scale feature fusion and suppress background noise. In addition, a parameter-free attention mechanism was integrated to adaptively emphasize critical features without increasing computational complexity. To improve detection of small defects, a composite loss function combining Normalized Wasserstein Distance and Intersection over Union was adopted. Experimental evaluation on a dataset of 6,000 AM images shows that YOLO-AMI attains a mean average precision (mAP@0.5) of 85.5%, precision of 87.1%, and recall of 83.2%, outperforming state-of-the-art models such as YOLOv8, YOLOv11, and RT-DETR-L. With an inference speed of 105.6 frames per second and a compact model size of 8.6 million parameters, the proposed approach achieves a favorable balance between accuracy and efficiency, providing a robust solution for intelligent online quality monitoring in Industry 4.0.

**Supplementary Information:**

The online version contains supplementary material available at 10.1038/s41598-026-44342-6.

## Introduction

As a revolutionary manufacturing technology, Additive Manufacturing (AM) has been widely applied in recent years across aerospace, automotive, biomedical, and mold manufacturing sectors, owing to its advantages such as rapid prototyping, high design freedom, and superior material utilization^1^.

However, due to various factors in the AM process—including equipment parameters, material flaws, and environmental fluctuations—the generation of defects is often inevitable; common defects include porosity, cracking, and warping, as well as “spaghetti”, “zits” and “stringing”, which are the focus of this paper^2^. From a computer vision perspective, “zits” and “stringing” present unique morphological challenges that render standard industrial sensors and generic detection models ineffective. Specifically, “zits” manifest as minute, semi-spherical protrusions with extremely low pixel occupancy (often occupying < 1% of the bounding box). Their topology closely mimics the surrounding layer textures, creating a low-contrast detection environment that is difficult to resolve without specialized feature fusion. Conversely, “stringing” appears as fine, high-aspect-ratio filaments spanning across void spaces. These delicate structures are prone to feature loss during the aggressive downsampling operations typical of standard CNNs.

These defects severely compromise the mechanical properties, dimensional accuracy, and service life of the products, posing significant risks to operational safety. Therefore, implementing efficient and precise defect detection is crucial for ensuring the quality of AM products. Traditional defect detection methods primarily rely on manual visual inspection or offline non-destructive testing (NDT) techniques, such as X-ray computed tomography (CT), ultrasonic testing, and thermal imaging^3^. Manual inspection is not only inefficient and time-consuming but also yields suboptimal results, as it is heavily influenced by subjectivity and the experience of the inspector^4^. Although offline NDT techniques offer high precision, they are hindered by expensive equipment and long detection cycles, making widespread adoption difficult; furthermore, as AM parts typically feature complex geometries, traditional offline methods often exhibit low robustness in specific scenarios^5^.

In recent years, with the advancement of artificial intelligence and the promotion of Industry 4.0, detection methods based on machine learning and machine vision have become increasingly popular in both academia and industry; in particular, deep learning technologies represented by Convolutional Neural Networks (CNNs) have seen widespread application in the field of online inspection^6^.Ting Sui & Junwen Wang^7^ proposed a dual-scale feature decoupling method that addresses the challenge of detecting minute scratch features on aluminum surfaces, which are easily obscured by high reflectivity and extrusion texture noise. Deep learning algorithms are frequently employed in academia and industry for detection and classification due to their powerful end-to-end capabilities; however, given the complexity of actual production environments where general-purpose models often fail to achieve high-precision detection, the selection of models and their optimization for specific scenarios have become critical. Guan-Qiang Wang et al.^8^ proposed a convolutional model for detecting surface defects (cracks, scratches) on steel strips, incorporating a Self-Attention Graph Convolution (SAGC) module that treats feature maps as node graphs; this approach allows for the inference and identification of defects even when they are intermittent, simultaneously improving the recall rate while significantly reducing parameters. Binyi Su et.al^9^ designed a novel Complementary Attention Network (CAN) embedded within Fast R-CNN, which utilizes a cascaded attention mechanism to achieve adaptive feature calibration; its core lies in deeply mining the aggregated features of Global Average Pooling (GAP) and Global Max Pooling (GMP) through convolutional layers, enabling the network to precisely focus on target regions while filtering redundant information. Doil Kim & Shuping Xiong^10^ proposed a method integrating coordinate attention mechanisms, Ghost convolution modules, transfer learning, and merged non-maximum suppression algorithms into the YOLOv8 model; deployed on a Jetson Xavier NX, the model achieved an average precision of 92.52% (mAP0.5) at 9.11 frames per second for personal protective equipment detection at the edge, marking a transition from manual to intelligent inspection. Filipe Pereira et al^11^ utilized an improved YOLOv5 algorithm, introducing Bot-Transformer and C2f. modules to detect yarn quality; the model achieved an average precision (mAP0.5) of 69.64% with a detection frame rate of 119 FPS, significantly reducing inspection costs while facilitating online non-destructive testing.

Beyond these specific manufacturing scenarios, deep learning has fundamentally revolutionized automated inspection and structural health monitoring (SHM) or non-destructive evaluation (NDE) in adjacent civil and structural engineering fields. For instance, in macro-scale monitoring, researchers have successfully deployed autonomous UAVs with ultrasonic beacon systems^12^ and multi-scale robotic approaches^13^ for precise concrete crack measurement. More recently, cutting-edge methodologies—such as Mamba-based segmentation integrated with laser metric calibration^14^, two-stage deep learning strategies for grouting defect detection using impact echo^15^, and hybrid XGBoost models for material strength prediction^16^—have demonstrated the immense potential of AI in robust defect evaluation. While these macro-scale SHM applications share the fundamental goal of intelligent anomaly detection and face similar deployment constraints (e.g., the need for high efficiency and edge-device adaptability) as our study, the sensing context and architectural choices distinctly differ. Macro-scale SHM often relies on multi-modal sensors (e.g., ultrasonic, impact echo, or UAV-mounted lasers) and heavy segmentation architectures to inspect large structures. Conversely, additive manufacturing inspection requires identifying micro-scale, visually subtle geometric defects in real-time using monocular vision. This distinct context necessitates the design of extremely lightweight yet highly sensitive network architectures tailored for minute defect feature extraction.

Convolutional Neural Networks (CNNs) are also being widely applied in the field of additive manufacturing defect detection. Tsan-Huang Fu & Dian-Li^17^ proposed a Multi-output Machine Learning real-time monitoring and closed-loop correction system, which achieved millisecond-level response and online repair of printing errors, drastically reducing the scrap rate. Hongliang Tuo et al.^18^ leveraged Infrared Thermography (IRT) and deep learning techniques to localize and measure internal defects in 3D-printed Carbon Fiber Reinforced Composites (CFRC); experiments demonstrated a model precision of 99.3% and a recall rate of 99.5%, achieving end-to-end intelligent inspection for 3D-printed products. Pandiyan et al.^19^ proposed a system that simultaneously acquires optical and acoustic signals, establishing a self-supervised learning model to detect defects in multi-material 3D printing; the authors designed a contrastive loss function that trains the model to pull photoacoustic signal pairs from the same compositional state closer in the feature space while pushing signals from different states apart. Jan Sher Akmal et al.^20^ used high-resolution X-ray Computed Tomography (XCT) data to train a deep learning model that establishes a precise mapping relationship between XCT-detected defect features and printing process parameters (e.g., laser power, scanning speed); focusing on titanium alloy (Ti-6Al-4 V), this study addressed the challenge of rapid detection in metal 3D printing. Yongqiang Zhang et al.^21^ utilized machine learning to learn the nonlinear relationship between sound speed gradients and beam deviation, correcting the delay of phased array received signals; this resolved issues in Functionally Graded Materials (FGM) where ultrasonic refraction or bending prevents traditional imaging algorithms from accurately focusing on defects, leading to missed detections or false positives.

Among numerous object detection algorithms, the YOLO (You Only Look Once) series has become a primary solution for visual inspection tasks due to its superior detection speed and high accuracy. From YOLOv1 to the present, the series has continuously evolved in terms of network structure and loss functions, forming a comprehensive family of YOLO models. Given that AM defect detection is characterized by high requirements for real-time performance and significant variations in defect morphology, YOLO is the optimal choice for this application due to its one-stage architecture, ultra-fast inference speed, and excellent global context perception capabilities^22^.

Although the YOLO series models possess outstanding detection capabilities, AM defects (such as zits) are often minute in size and varied in morphology, features which pose significant challenges to the accuracy, localization ability, and robustness of object detection models^23^. Crucially, the layer-by-layer deposition process in 3D printing creates complex surface textures characterized by periodic aliasing and striations. Standard deep learning models, which typically rely on aggressive downsampling to extract semantic features, often struggle to differentiate between these regular texture patterns and minute, irregular defects. Consequently, generic networks frequently misinterpret the background layer lines as noise or fail to resolve small defects hidden within the textured ridges. This study utilizes YOLOv10^24^as the baseline model and establishes the YOLO-AMI model specifically for AM defect detection by optimizing the neck network, attention mechanisms, and loss functions through the introduction of the Asymptotic Feature Pyramid Network (AFPN) structure, SimAM (a parameter-free attention mechanism), and a composite loss function combining Normalized Wasserstein Distance (NWD) and Intersection over Union (IoU). Through a series of comparative and ablation experiments, this study verifies the effectiveness of the model, aiming to provide a high-precision, high-efficiency online detection solution for the AM field and to drive the quality control of additive manufacturing toward intelligence and automation.

## Methodology

**Fig. 1:**
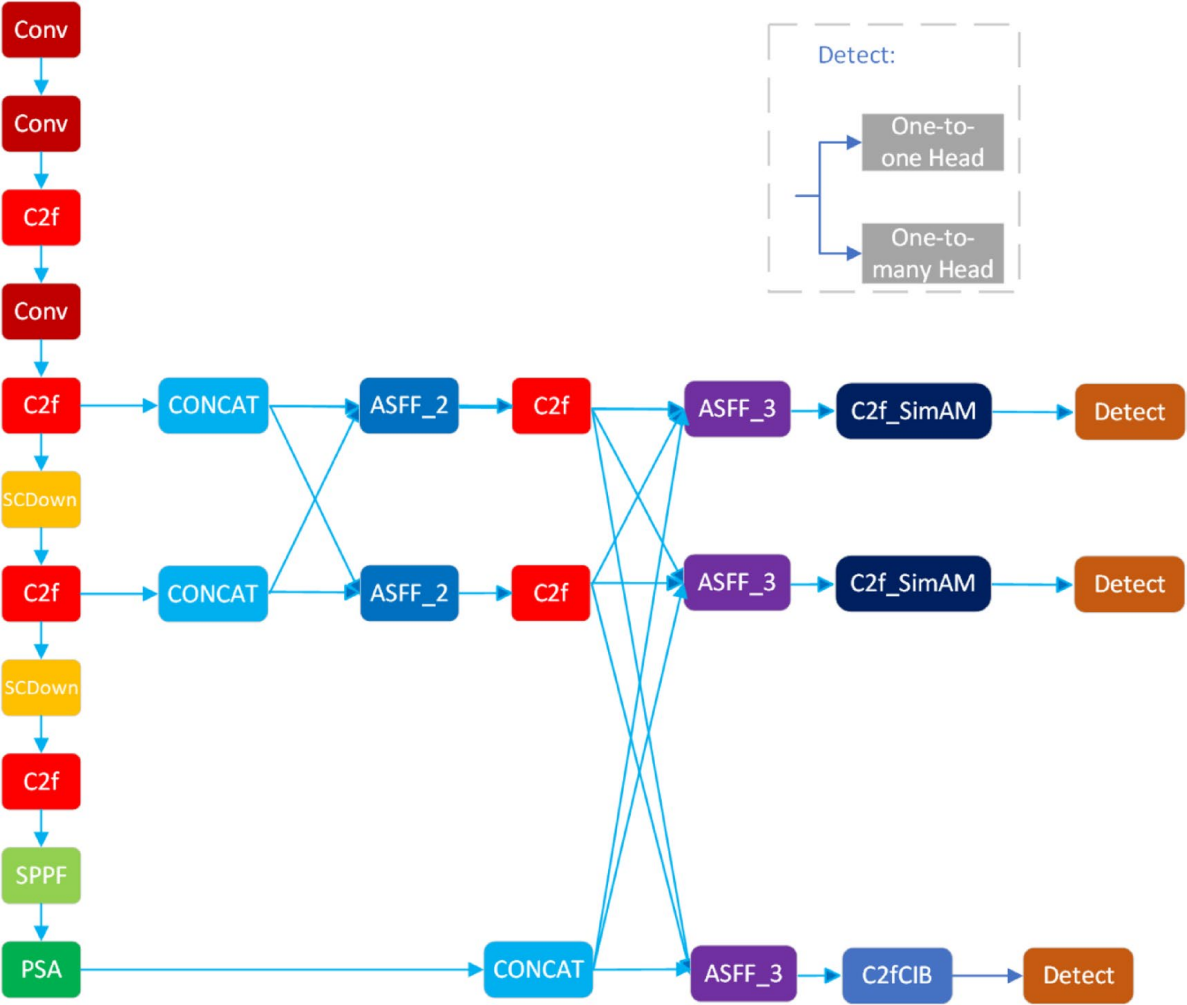
Overall framework of the proposed YOLO-AMI model.

To specifically address the limitations encountered by YOLOv10 in detecting additive manufacturing defects—namely insufficiency in multi-scale fusion, weak feature extraction, and the low localization accuracy of small objects—this study proposes an improved model, YOLO-AMI. The overall architecture of the proposed model is illustrated in Fig. 1. The specific enhancements include: (I) Reconstruction of the neck network: The Asymptotic Feature Pyramid Network (AFPN) replaces the original neck structure, achieving more efficient multi-scale fusion and effectively suppressing noise interference, thereby generating fused features with enhanced discriminative power. (II) Integration of attention mechanisms: A simple attention mechanism (SimAM) is introduced to adaptively extract and enhance features without significantly increasing computational complexity (FLOPs), enabling the model to focus on critical information and improving its representational capability. (III) Optimization of the loss function: To address the challenges of bounding box regression in small object detection, the original loss function is replaced by a hybrid strategy combining Normalized Wasserstein Distance (NWD) and Intersection over Union (IoU). This composite loss aims to significantly boost localization accuracy for small defects while preserving robustness for standard-sized targets.

### Overview of the baseline YOLOv10

**Fig. 2:**
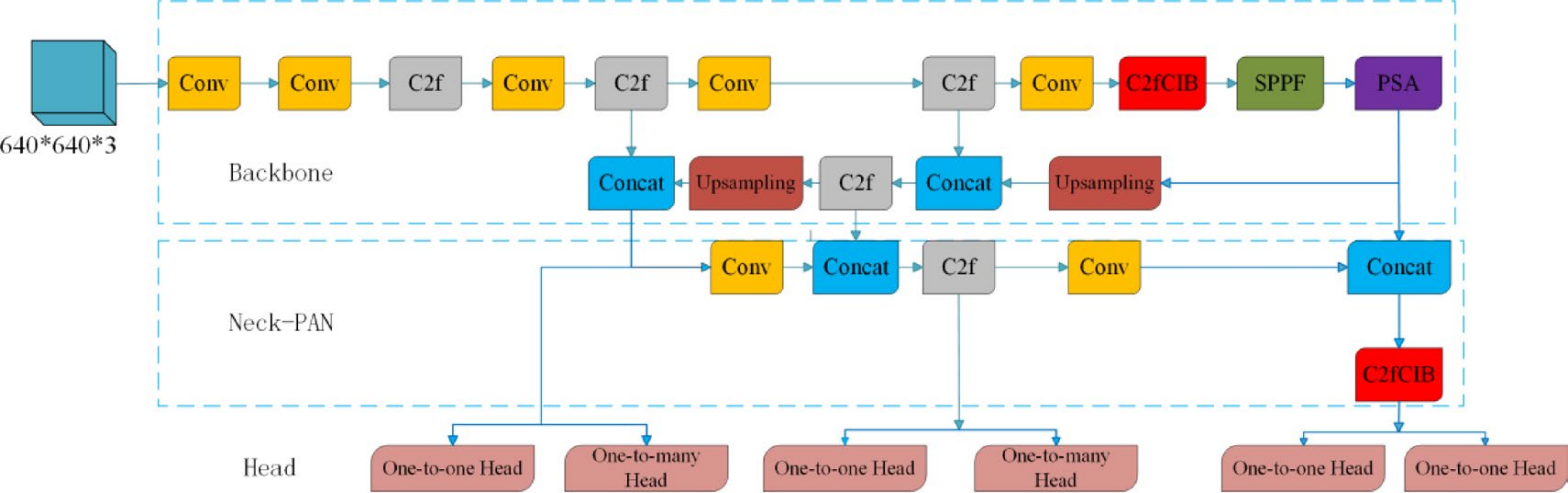
Schematic of the baseline YOLOv10 architecture.

Proposed by a research team from Tsinghua University in 2024, YOLOv10 is a lightweight one-stage detector within the YOLO series designed to further enhance inference efficiency while maintaining high detection accuracy; compared to previous YOLO models, YOLOv10 incorporates the following improvements: (I) In the backbone architecture, YOLOv10 prunes redundant convolutional channels to reduce inference computational costs, thereby facilitating deployment on resource-constrained platforms such as GPUs and embedded systems. (II) Elimination of Non-Maximum Suppression (NMS) and the introduction of a consistent dual assignment strategy. Given that NMS is prone to erroneously suppressing true targets in dense small-object scenarios, this strategy enables more robust handling of positive/negative sample assignment and label matching during the training phase, thereby effectively enhancing the recall rate and localization stability for small objects. By assigning both one-to-one and one-to-many labels to each target during the training process, this strategy resolves the redundancy issues typically managed by NMS, consequently significantly reducing inference latency. (III) In the neck architecture, YOLOv10 eliminates unnecessary computations and optimizes the Spatial-Channel Decoupled Downsampling and Rank-Guided modules, thereby achieving highly compressed fused features and attaining equivalent or superior feature representation capabilities with reduced parameter counts and computational loads. The overall architecture of the YOLOv10 is illustrated in Fig. 2.

Based on YOLOv10, this study targets common defects in additive manufacturing (spaghetti, zits, and stringing) and modifies the model by enhancing the neck network structure, optimizing the loss function, and introducing attention mechanisms to achieve superior defect recognition performance.

### Multi-scale feature fusion based on AFPN

Defects in 3D-printed products are typically very small and have complex spatial distributions. To improve detection, this study modifies YOLOv10’s neck by adopting the Asymptotic Feature Pyramid Network (AFPN)^25^.

AFPN is a multi-scale feature-fusion architecture that uses direct interaction paths and an asymptotic fusion strategy. By avoiding up- and down-sampling in the fusion path, it better preserves the original feature information.

AFPN first fuses two adjacent low-level features and then progressively integrates higher-level features. This asymptotic, adjacent-first fusion reduces the large semantic gaps that can occur between non-adjacent feature levels. This asymptotic fusion strategy is particularly advantageous for mitigating the interference of layer-by-layer aliasing common in 3D printed surfaces. Unlike standard FPNs that abruptly fuse deep semantic features with shallow patterns, AFPN integrates features gradually. This prevents the loss of high-frequency spatial details caused by excessive downsampling, allowing the network to effectively learn the regularity of layer striations and distinguish them from the irregular morphological anomalies of defects. Furthermore, this capability to preserve low-level spatial details is critical for counteracting the potential information loss caused by resizing images to 640 $\times$ 640. By maintaining direct interaction with shallow feature layers, AFPN ensures that the structural integrity of extremely minute defects like “zits” (which may occupy only 2–5 pixels) is preserved and propagated, preventing them from vanishing during the feature extraction process.

**Fig. 3:**
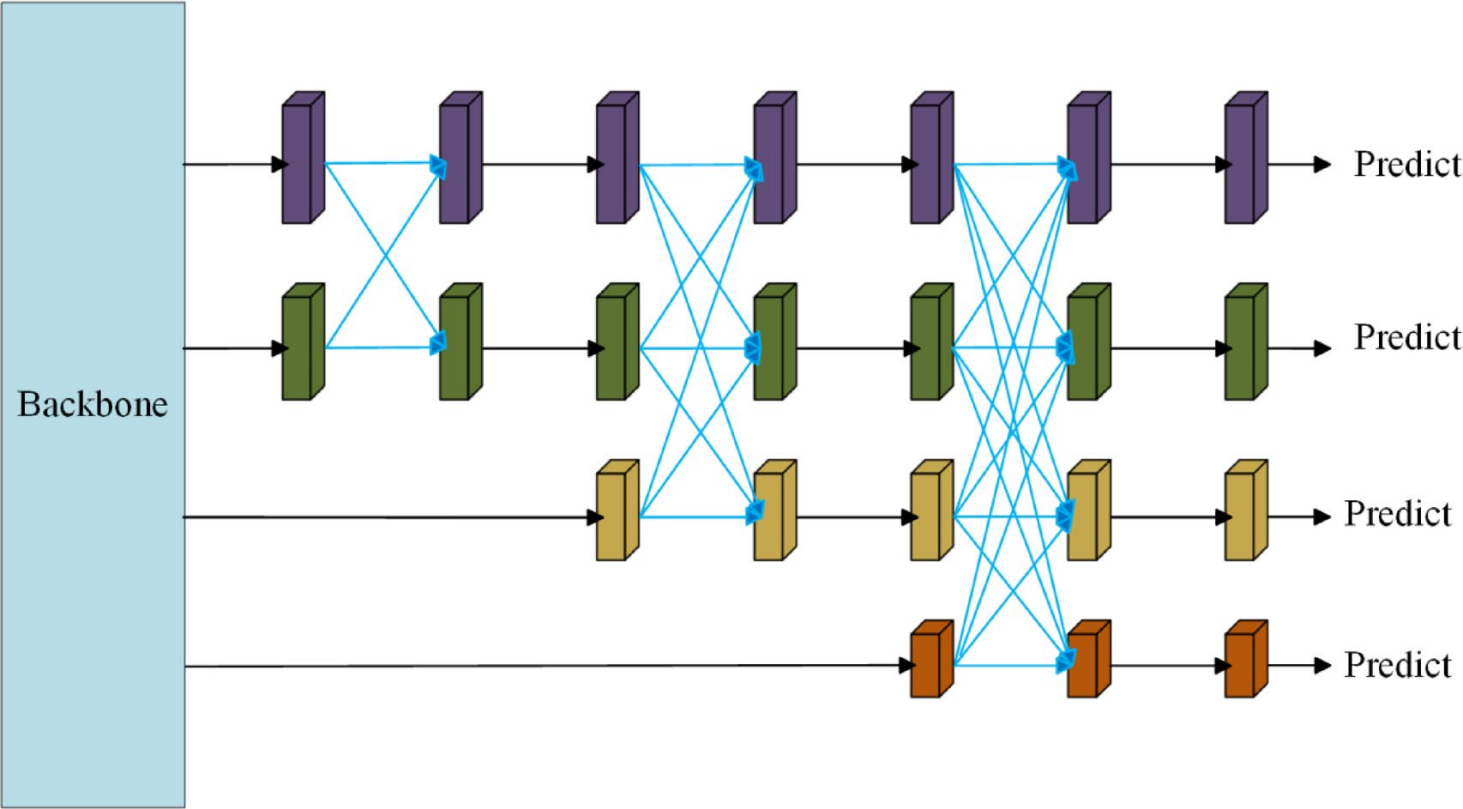
Structure of the asymptotic feature pyramid network (AFPN).

 The AFPN structure (see Fig. 3) fuses features from deep to shallow in a top-down manner. Its workflow is: (I) align channel dimensions of backbone outputs with 1 × 1 convolutions; (II) perform same-resolution fusion via multi-level progressive fusion using adaptive spatial pooling; and (III) produce a multi-scale feature pyramid as the output.

Adaptive Spatial Feature Fusion (ASFF) was integrated into the multi-level fusion process. ASFF selectively filters conflicting information while adding negligible spatial inference overhead, thereby enhancing scale invariance and the representational capacity of multi-level features. The fusion design supports both two-level and three-level fusion. For three-level feature fusion, denote ${{X}_{ij}}^{(n\to l)}$ as the feature vector propagated from level n to level l, and ${{y}_{ij}}^{(l)}$ the resulting fused feature vector at level l. The adaptive fusion is then defined as:

1 $$\begin{array}{*{20}c} {y_{ij}^{\left( l \right)} = \alpha_{ij}^{\left( l \right)} \cdot x_{ij}^{{\left( {1 \to l} \right)}} + \beta_{ij}^{\left( l \right)} \cdot x_{ij}^{{\left( {2 \to l} \right)}} + \gamma_{ij} \cdot x_{ij}^{{\left( {3 \to l} \right)}} } \\ \end{array}$$

Here, ${\upalpha }_{{{\mathrm{ij}}}}^{{(1)}}$,${\upbeta }_{{{\mathrm{ij}}}}^{(1)}$ and ${\mathrm{y}}_{{{\mathrm{ij}}}}^{(1)}$ denote the spatial weight assigned to the three feature levels at position (i,j,), subject to the constraint:

2 $$\begin{array}{*{20}c} {\alpha_{ij}^{\left( l \right)} + \beta_{ij}^{\left( l \right)} + y_{ij}^{\left( l \right)} = 1} \\ \end{array}$$

This formulation inherently accommodates variations in the number of feature maps across different levels, thereby generalizing the spatial fusion module to handle any given number of inputs.

**Fig. 4:**
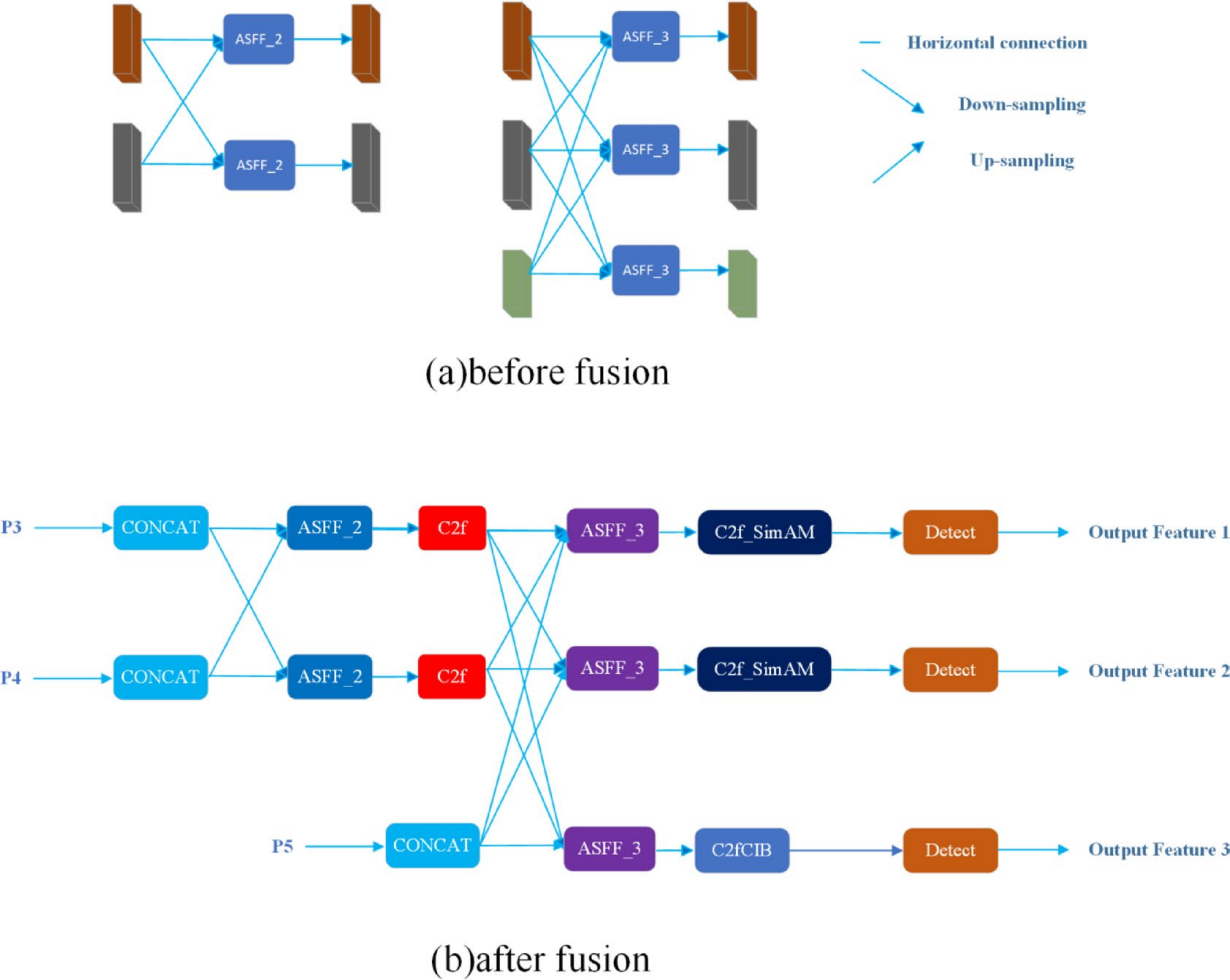
The AFPN-based neck network structure after fusion.

The proposed neck network, built on AFPN and ASFF, performs seamless fusion of the three backbone outputs to facilitate accurate detection of small, spatially complex defects in 3D-printed products (see Fig. 4).

### Feature enhancement with parameter-free attention(SimAM)

SimAM (Simple, Parameter-Free Attention Module)^26^ is a parameter-free attention mechanism that does not increase network complexity. Standard YOLO architectures utilize deep convolutional backbones that rely heavily on edge detection kernels. In the specific context of 3D printing, the dense, periodic layer lines (texture noise) generate strong, high-frequency edge responses that propagate through the network. These background “noise” activations often overwhelm the weaker feature signatures of minute defects like zits, leading to false negatives. SimAM addresses this by calculating 3-D attention weights based on the energy function of neurons**.** Inspired by the neuroscience concept of “spatial suppression”, SimAM defines an energy function to quantify the importance of individual neurons. Importance weights are thus assigned to each spatial location (and channel) within the feature map. This approach improves performance while adding zero parameters and imposing negligible computational overhead. The energy function *et*(*) is defined as:

3 $$\begin{array}{*{20}c} {e_{t} \left( {w_{t} ,b_{t} ,y,x_{i} } \right) = \left( {y_{t} - \hat{t}} \right)^{2} + \frac{1}{M - 1}\mathop \sum \limits_{i = 1}^{M - 1} \left( {y_{0} - \hat{x}_{i} } \right)^{2} } \\ \end{array}$$

where:

4 $$\left\{ {\begin{array}{*{20}l} { \hat{t} = w_{t} t + b_{t} } \hfill \\ {\hat{x}_{i} = w_{t } x_{i} + b_{t} } \hfill \\ \end{array} } \right.$$

In Eq. (4),t denotes the target neuron and ${x}_{i}$ denotes other neurons of the input feature tensor $X\in {R}^{c\times h\times w}$. Here c, h and w denote channel count, height, and width, respectively. The spatial index is written as i, and $M=h\times w$ quals the number of neurons per channel. Parameters ${w}_{t}$ and ${b}_{t}$ refer to the weight and bias of a linear transformation applied in the derivation. y, ${y}_{t}$ and ${y}_{0}$ are scalars, binary labels ${y}_{t}$ and ${y}_{0}$ are employed during computation, specifically defined as $y_{t} = 1\; {\mathrm{and}}\;y_{0} = - 1$. The final energy function is expressed as:

5 $$\begin{array}{*{20}c} {e_{t} \left( {w_{t} ,b_{t} ,y,x_{i} } \right) = + \frac{1}{M - 1}\mathop \sum \limits_{i = 1}^{M - 1} \left( { - 1 - w_{t } x_{i} + b_{t} } \right)^{2} + \left( {1 - w_{t } x_{i} + b_{t} } \right)^{2} + \lambda w_{i}^{2} } \\ \end{array}$$

In Eq. (5), $\uplambda$ represents the regularization coefficient (which is empirically set to $1\times {10}^{-4}$ in our experiments to prevent division by zero and stabilize the energy calculation), and ${w}_{i}$ denotes the transformation weight of the i-th neuron. Solving Eq. (5) yields the following:

6 $$\left\{ {\begin{array}{*{20}l} { w_{t} = \frac{{2\left( {t - \mu_{t} } \right)}}{{\left( {t - \mu_{t} } \right)^{2} + 2\sigma_{t}^{2} + 2\lambda }}} \hfill \\ {b_{t} = - \frac{1}{2}\left( {t + \mu_{t} } \right)w_{t} ,} \hfill \\ \end{array} } \right.$$

In Eq. (6),

7 $$\left\{ {\begin{array}{*{20}l} { \mu_{t} = \frac{1}{M - 1}\sum ix_{i} } \hfill \\ {\sigma_{t} = \sqrt {\frac{1}{M - 1}\sum i\left( {x_{i} - \mu_{t} } \right)^{2} } } \hfill \\ \end{array} } \right.$$

Substituting ${w}_{t}$ and ${b}_{t}$ from Eq. (6) into Eq. (7) yields the minimum energy calculation formula:

8 $$\begin{array}{*{20}c} {e_{t}^{*} = \frac{{4\left( {\delta^{2} + \lambda } \right)}}{{\left( {t - \hat{\mu }} \right)^{2} + 2\hat{o}^{2} + 2\lambda }}} \\ \end{array}$$

In Eq. (8), by substituting the mean $\widehat{\mu }=\frac{1}{M}{\sum }_{i=1}^{M}{x}_{i}$ for ${\mu }_{i}$, and the variance ${\widehat{o}}^{2}=\frac{1}{M-1}{\sum }_{i=1}^{M}{({x}_{i}-\widehat{\mu })}^{2}$ for ${\sigma }_{i}^{2}$, the activity level of the target neuron T s shown to be inversely proportional to the energy ${e}_{t}^{*}$. Thus, a lower ${e}_{t}^{*}$ indicates greater importance in visual processing. Accordingly, the importance of each neuron is given by $\frac{1}{{e}_{t}^{*}}$. Feature enhancement is then performed using Eq. (9):

9 $$\begin{array}{*{20}c} {\tilde{X} = sigmoid\left( \frac{1}{E} \right) \odot X} \\ \end{array}$$

where,$X$ denotes the input feature tensor, E denotes the aggregation of ${e}_{t}^{*}$ across all channels and spatial dimensions, and the sigmoid function suppresses excessively large values in* E*. Crucially, this energy-based attention mechanism compensates for the potential loss of spatial fidelity caused by image resizing. By assigning higher importance weights to neurons exhibiting spatial anomalies, SimAM amplifies the weak feature signals of small, low-resolution zits against the background, ensuring they remain detectable even when their pixel footprint is minimized by downsampling.

**Fig. 5:**
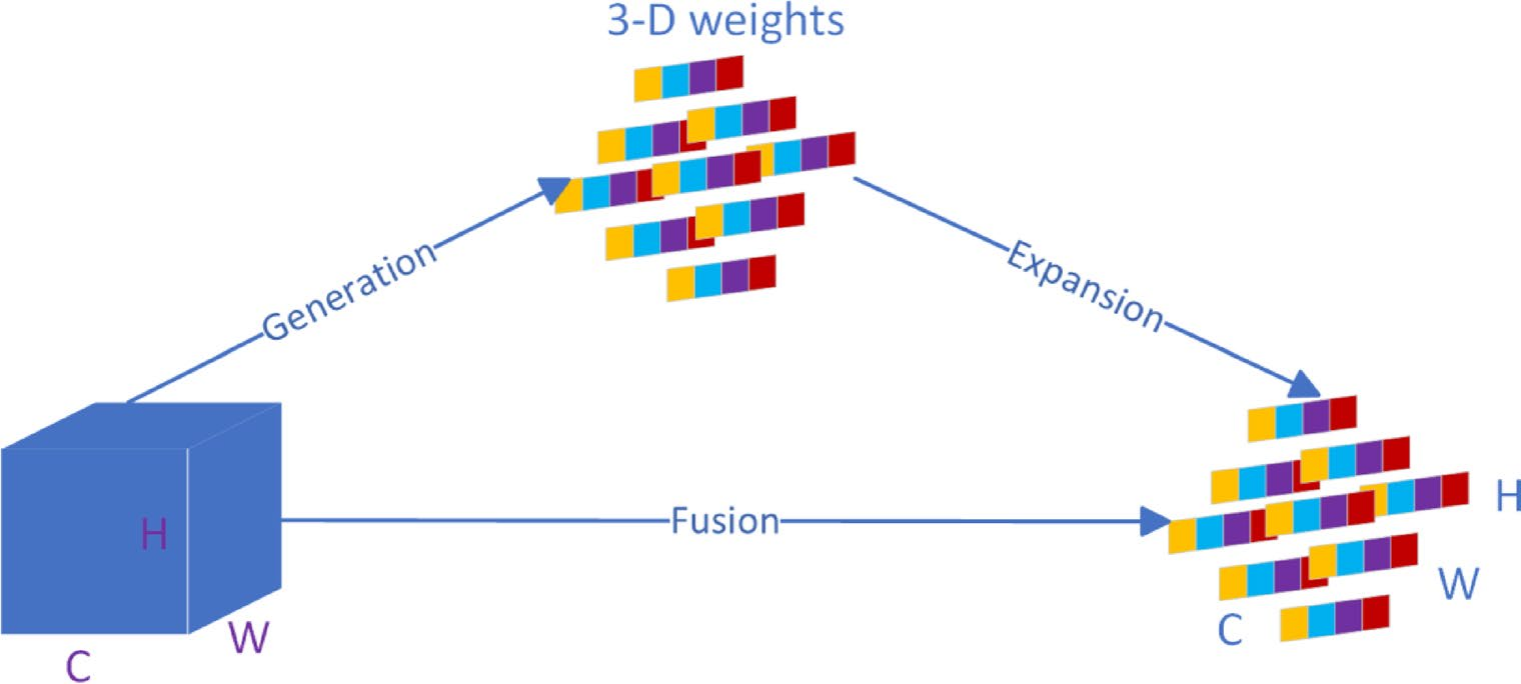
Diagram of the SimAM parameter-free attention module.

**Fig. 6:**
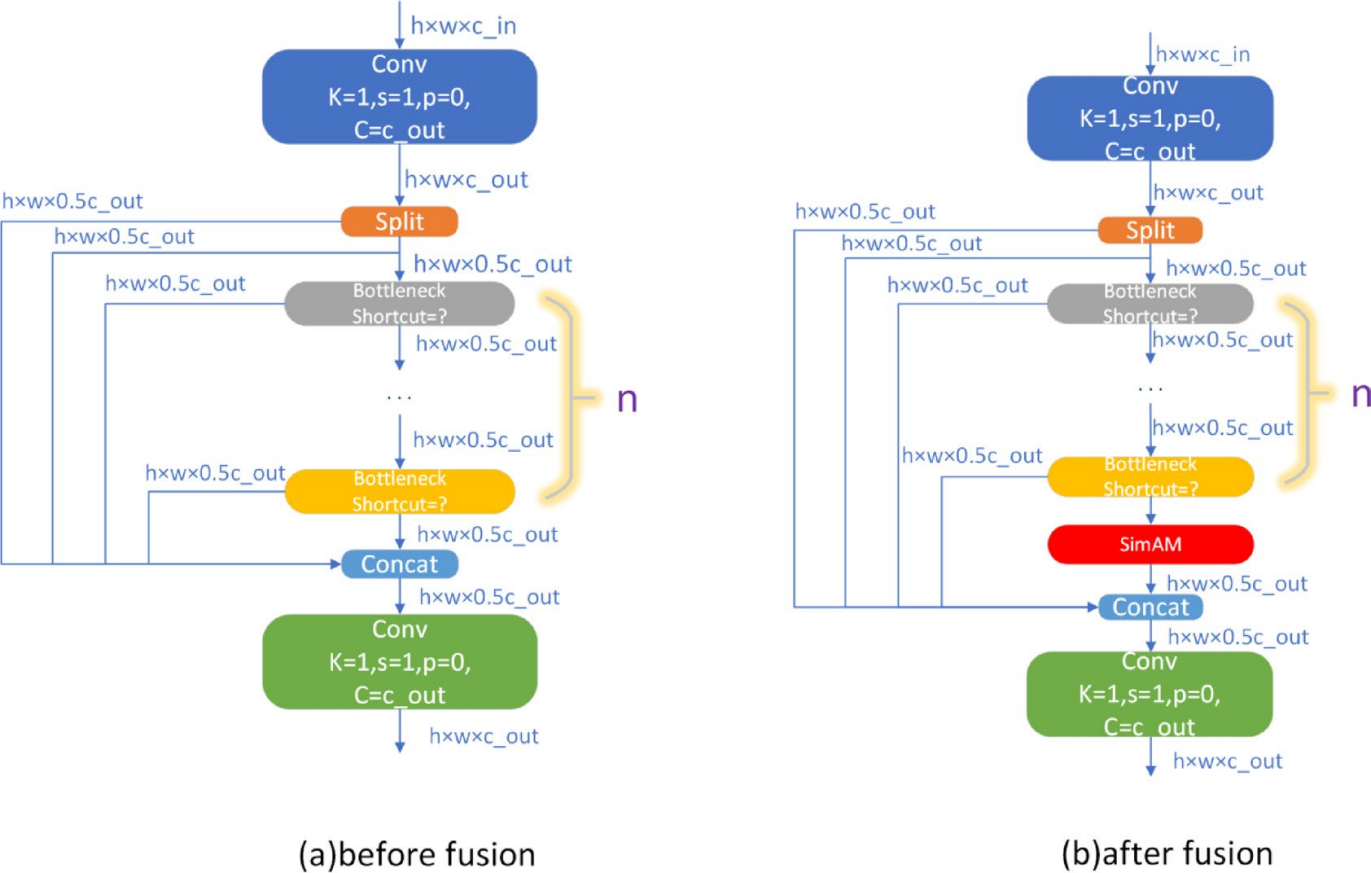
Structure of the C2f module in the backbone.

Figure 5 illustrates the feature-map processing of SimAM. A feature map of dimensions $C\times H\times W$ processed by 3-D weights, expanded to match the input dimensions, and fused with the input to produce weighted output features. Figure 6 structure of the C2f. module in the backbone.

Furthermore, this energy-based metric provides inherent robustness against environmental noise. Since lighting artifacts and surface reflections typically manifest as global or low-frequency intensity shifts, SimAM—which focuses on local neuronal singularity—effectively suppresses these irrelevant visual cues. This ensures that the network attends strictly to the morphological structure of defects rather than reacting to lighting variations, as empirically verified in the feature visualization results.

### Improved loss function via normalized Wasserstein distance

When preparing 3D-printing defect data samples, it was observed that small target sizes and concealed textures can cause loss of critical features during initial feature extraction, reducing object detection accuracy.

**Fig. 7:**
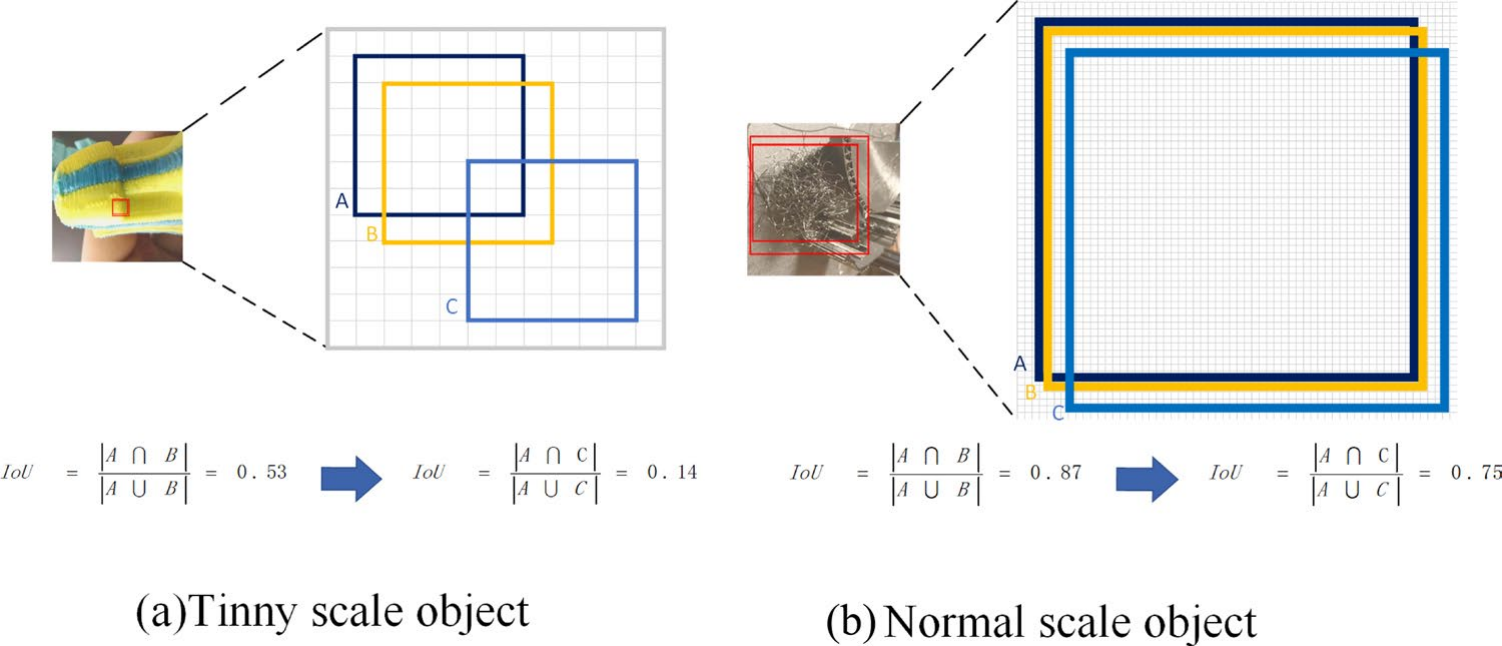
IoU sensitivity analysis.

Traditional Intersection over Union (IoU) metrics, owing to their geometric nature, often drop rapidly to zero or become highly sensitive to small localization errors when applied to small objects. Figure 7 show detection bounding boxes for targets of size 6 $\times$ 6 pixels and 55 $\times$ 55 pixels, respectively. A denotes the ground-truth box, while B and C denote predicted boxes with diagonal errors of 1 pixel and 4 pixels, respectively. For the same absolute deviation, small targets receive a much larger penalty than large targets; this imbalance negatively affects label assignment and degrades detection performance.

To address this problem, this study adopts the Normalized Wasserstein Distance (NWD) loss function^27^. Experiments on public datasets such as COCO and VisDrone show that NWD substantially improves small-object detection accuracy. NWD represents rectangular bounding boxes (bbox) as two-dimensional Gaussian distributions and computes the second-order Wasserstein distance between them to capture differences in location and scale. The resulting distance is converted into a similarity score in the range $\left(0,\left.1\right]\right.$ via exponential normalization. Compared with IoU, NWD is less sensitive to object-size variations and thus substantially increases robustness for detecting minute targets.

Specifically, to obtain a distribution-based bounding-box similarity metric, NWD models each bounding box $B = \left( {cx,cy,w,h} \right)$ as a two − dimensional Gaussian distribution ${{\rm N}} = \left( {\mu ,\sum } \right)$. The mean vector $\mu = \left( {cx,cy} \right)$ denotes the object center, and the covariance matrix $$\sum = \left( {\begin{array}{*{20}c} {\left( {\frac{\omega }{2}} \right)^{2} } & 0 \\ 0 & {\left( \frac{h}{2} \right)^{2} } \\ \end{array} } \right)$$ encodes the spatial extent. Within this probabilistic framework, the relationship between two bounding boxes A and B is quantified by the second-order Wasserstein distance between their corresponding Gaussian distributions ${{\rm N}}_{a}$ and ${{\rm N}}_{b}$ expressed as Eq. (9).

10 $$\begin{array}{*{20}c} {W_{2}^{2} \left( {{{\rm N}}_{a} ,{{\rm N}}_{b} } \right) = \left| {\left| {\mu_{a} - \mu_{b} } \right|} \right|^{2} + Tr\left( {\sum_{a} + \sum_{b} } \right) - 2\sum_{a}^{\frac{1}{2}} \sum_{b}^{\frac{1}{2}} } \\ \end{array}$$

The above formula can be simplified to a more compact form (see Eq. (11))

11 $$\begin{array}{*{20}c} {W_{2}^{2} \left( {{{\rm N}}_{a} ,{{\rm N}}_{b} } \right) = \left| {\left| {\mu_{a} - \mu_{b} } \right|} \right|^{2} + \left| {\left| {\sum_{a}^{\frac{1}{2}} + \sum_{b}^{\frac{1}{2}} } \right|} \right|_{F}^{2} } \\ \end{array}$$

For Gaussian distributions ${{\rm N}}_{a}$ and ${{\rm N}}_{b}$ derived from bounding boxes $A=\left(c{x}_{a},c{x}_{a},{\omega }_{a},{h}_{a}\right)$ and $B=\left(c{x}_{b},c{x}_{b},{\omega }_{b},{h}_{b}\right)$ the expression admits a further simplification, shown in Eq. (12):

12 $$\begin{array}{*{20}c} {W_{2}^{2} \left( {{{\rm N}}_{a} ,{{\rm N}}_{b} } \right) = \left| {\left| {\mu_{a} - \mu_{b} } \right|} \right|^{2} + \left| {\left| {\left( {cx_{a} ,cy_{a} ,\frac{{\omega_{a} }}{2},\frac{{h_{a} }}{2}} \right)^{T} ,\left( {cx_{a} ,cy_{a} ,\frac{{\omega_{a} }}{2},\frac{{h_{a} }}{2}} \right)^{T} } \right|} \right|_{2}^{2} } \\ \end{array}$$

To obtain a similarity metric on a unified scale, an exponential normalization is applied, defining the normalized distance as Eq. (13):

13 $$\begin{array}{*{20}c} {NWD\left( {A,B} \right) = exp\left( { - \frac{{\sqrt {W^{2} \left( {{{\rm N}}_{a} {{\rm N}}_{b} } \right)} }}{C}} \right)} \\ \end{array}$$

where, $C$ denotes a normalization constant, in our experiments, $C$ is empirically set to 12.8 based on the average scale of the defects in our 3D-printing dataset. This approach is robust to geometric variations in minute objects and can therefore replace IoU in multiple stages, including positive/negative sample assignment, non-maximum suppression (NMS), and regression loss computation. In the loss function, NWD and IoU are combined linearly to form a composite supervisory signal:

14 $$\begin{array}{*{20}c} {L = \alpha \cdot \left( {1 - NWD} \right) + \left( {1 - \alpha } \right) \cdot \left( {1 - IoU} \right)} \\ \end{array}$$

In this expression, $\alpha$ is the weighting coefficient. To determine the optimal value for $\alpha$, we conducted a systematic sensitivity analysis with values ranging from 0.1 to 0.9. As presented in Table 1.Different $\alpha$ Value Profermance Table 1, the detection performance peaks when $\alpha$ = 0.7. This value offers the best trade-off: lower $\alpha$ values (< 0.5) rely too heavily on IoU and miss small defects, while higher values (> 0.8) reduce localization precision. Accordingly, $\alpha = 0.7$; accordingly, $\alpha = 0.7$ is adopted, which simplifies Eq. (14) to Eq. (15).

15 $$\begin{array}{*{20}c} {L = 0.7 \cdot \left( {1 - NWD} \right) + 0.3 \cdot \left( {1 - {\mathrm{IoU}}} \right)} \\ \end{array}$$

**Table 1:** Different $\alpha$ value profermance.

$\alpha$(NWD Weight)	Precision (%)	Recall (%)	mAP@0.5 (%)	mAP@0.5:0.95%
0.1	83.5	78.9	81.5	48.8
0.3	84.2	80.1	82.4	49.6
0.5	85.0	81.2	83.3	50.5
0.6	85.4	81.8	83.9	51.0
**0.7**	**85.8**	**82.3**	**84.2**	**51.4**
0.8	85.3	81.7	83.7	50.8
0.9	84.6	80.5	82.8	49.9

Bold values indicate the optimal performance metrics achieved.

Consequently, the final loss expression becomes Eq. (16).

16 $$\begin{array}{*{20}c} {L = \frac{1}{N}\mathop \sum \limits_{i = 1}^{N} 0.7 \cdot \left( {1 - NWD_{i} } \right) + 0.3 \cdot \left( {1 - {\mathrm{IoU}}_{i} } \right)} \\ \end{array}$$

In the above, N denotes the number of detection boxes.

### Dataset construction and preprocessing

To construct the experimental portion of the dataset, 3D printing experiments were conducted using a Flashforge Adventurer 3 Pro Fused Deposition Modeling (FDM) printer. We utilized standard commercial Polylactic Acid (PLA) filaments (1.75 mm diameter). The printing parameters were set to standard manufacturer-recommended profiles to simulate representative manufacturing conditions: a nozzle diameter of 0.4 mm, a layer height of 0.2 mm, a printing temperature of 210 °C, a bed temperature of 50 °C, and a deposition speed of 50 mm/s. Image acquisition was performed using a high-resolution smartphone camera (12 MP) under standard ambient lighting conditions (a mix of natural and artificial light) without specialized auxiliary illumination. This setup was intentionally chosen to simulate realistic, low-cost manufacturing environments where controlled studio lighting is often unavailable, thereby introducing natural variations in brightness and shadow to enhance model robustness.

Data were obtained via experimental procedures and targeted web collection strategy**.** The web-collected images were rigorously aggregated from public open-source repositories (e.g., GitHub, Kaggle) and excavated from community-driven technical platforms (such as 3D printing troubleshooting archives). To ensure that these diverse sources are representative of valid controlled manufacturing environments, a strict manual curation process was implemented. We specifically filtered for images exhibiting high morphological fidelity to FDM defects while excluding low-resolution samples or those originating from non-relevant processes (e.g., SLA). This strategy introduces real-world variability, ensuring the dataset encompasses the long-tail distribution of defect appearances beyond the limited scope of laboratory settings. With three common additive-manufacturing defect types—spaghetti, zits, and stringing—selected for labeling and dataset preparation. Sample images were uniformly resized to fixed dimensions (640 × 640 pixels). LabelImg was used to annotate bounding boxes and assign labels, and the annotations were saved in PASCAL VOC format. Subsequently, to align with the standard input requirements of the YOLO architecture, these annotation files were batch-converted into the standard YOLO format (TXT). To improve model robustness and generalization, data augmentation was applied, including random rotation, scaling, and adjustments to color and grayscale. Specifically, to mitigate potential bias arising from specific material properties (e.g., color, reflectivity, or sheen of the filament), we implemented aggressive photometric distortions, including random changes in hue, saturation, brightness, and contrast (Color Jitter). Furthermore, the inclusion of diverse web-collected images introduces high variability in material appearance. These strategies force the model to focus on the inherent morphological features of the defects (such as the structural topology of zits and stringing) rather than relying on superficial color or texture cues, thereby ensuring robustness across different printing materials. Given the inherent challenges of detecting minute targets and the severe class imbalance present in the dataset, deep learning models can occasionally exhibit training variance. To rigorously ensure the statistical reliability and generalizability of our conclusions, the proposed YOLO-AMI model was evaluated across three independent experimental runs initialized with different random seeds.

**Fig. 8:**
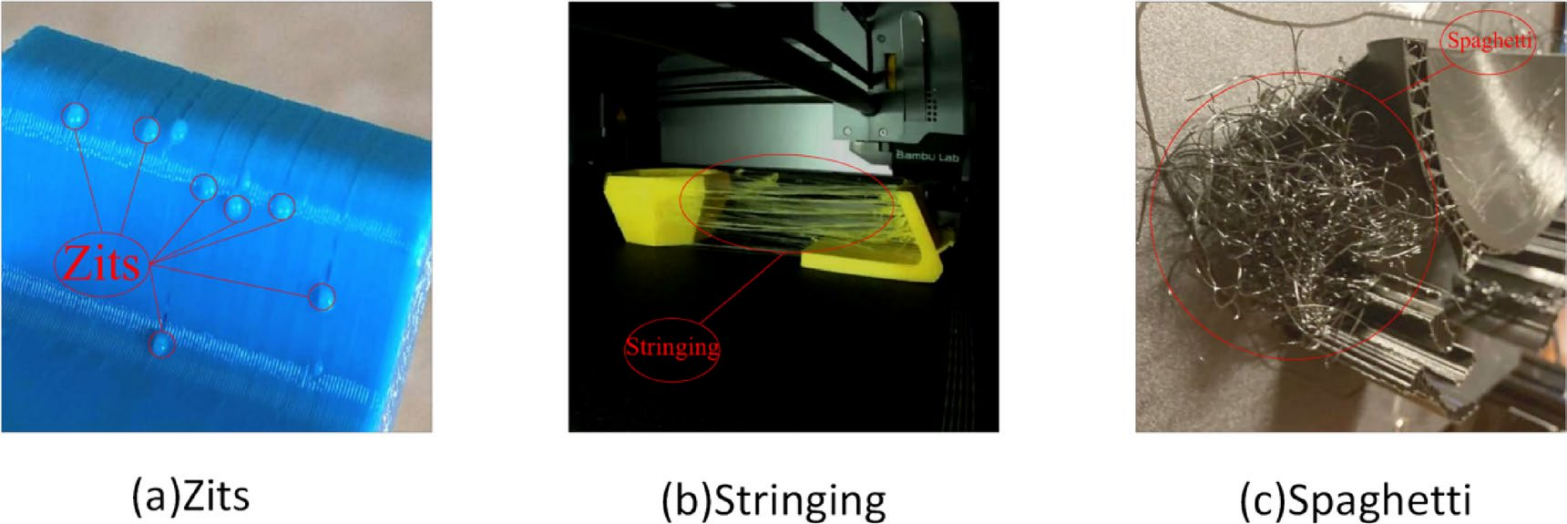
Typical defect examples.

The final dataset contains 6,000 images, split into training, validation, and test sets at an 8:1:1 ratio, yielding 4,800 training images, 600 validation images, and 600 test images. To rigorously prevent potential data leakage and ensure a robust evaluation of model generalization, this split was strictly performed at the print-job (i.e., sequence) level rather than the individual frame level. Specifically, all images originating from a single continuous print job were exclusively assigned to only one of the subsets (training, validation, or testing), ensuring the model is evaluated on entirely unseen print processes. Figure 8 shows representative samples of the three defect types studied in this paper. The detailed hyperparameters and application probabilities for all augmentation strategies are summarized in Table 2.

**Table 2:** Detailed hyperparameters and probabilities for data augmentation.

Augmentation method	Parameter/probability	Description
Mosaic	1.0 (Prob)	Combines 4 training images into 1
MixUp	0.1 (Prob)	Blends two images with different transparencies
HSV-Hue	0.015 (Fraction)	Random adjustments to image hue
HSV-saturation	0.7 (Fraction)	Random adjustments to saturation
HSV-value	0.4 (Fraction)	Random adjustments to brightness/value
Rotation	$\pm$ 10.0 (Degrees)	Random image rotation
Scale	$\pm$ 0.5 (Gain)	Random scaling (zoom in/out)
Flip left–right	0.5 (Prob)	Horizontal flipping

## Experiments and discussion

The experiment used the PyTorch 2.0.1 framework in a Python 3.9.21 environment to build the detection model for additive-manufacturing products. The experimental platform ran Windows 10 with an NVIDIA RTX3060 GPU.

### Experimental setup and evaluation metrics

To evaluate detection accuracy, Precision, Recall, and Mean Average Precision (mAP) were selected as metrics.

Precision measures the probability that samples predicted as positive are actually positive; it emphasizes prediction exactness and is computed as:

17 $$\begin{array}{*{20}c} {P = \frac{{{\mathrm{TP}}}}{{{\mathrm{TP}} + {\mathrm{FP}}}}} \\ \end{array}$$

where, TP is the number of true positives and FP is the number of false positives.

Recall measures the probability that actual positive samples are correctly detected; it reflects detection coverage and is computed as:

18 $$\begin{array}{*{20}c} {R = \frac{{{\mathrm{TP}}}}{{{\mathrm{TP}} + {\mathrm{FN}}}}} \\ \end{array}$$

where, FN denotes the number of false negatives (positive samples missed by the model).

Mean Average Precision (mAP) is the arithmetic mean of Average Precision (AP) values across classes; it reflects overall recognition performance, with higher mAP indicating better detection. The calculation is:

19 $$\begin{array}{*{20}c} {mAP = \frac{{\mathop \sum \nolimits_{c = 1}^{c} {\mathrm{A}}P_{C} }}{{\mathrm{C}}}} \\ \end{array}$$

where, $\sum_{c=1}^{c}\mathrm{A}{P}_{C}$ is the sum of AP values for all classes and C is the total number of classes.

### Implementation details and dataset

During model training, parameter tuning was conducted to improve model robustness. Table 3 summarizes the parameter settings.

**Table 3:** Training parameter setting.

Parameter	Value	Parameter	Value
Epochs	300	initial_lr (lr0)	0.01
Patience	50	Optimizer	Auto
batch_size	8	Momentum	0.937
imgsz	640	weight_decay	0.0005
box_loss_gain	7.5	warmup_momentum	0.8
cls_loss_gain	0.5	dfl_loss_gain	1.5
final_lr (lrf)	0.01	warmup_epochs	3.0

**Fig. 9:**
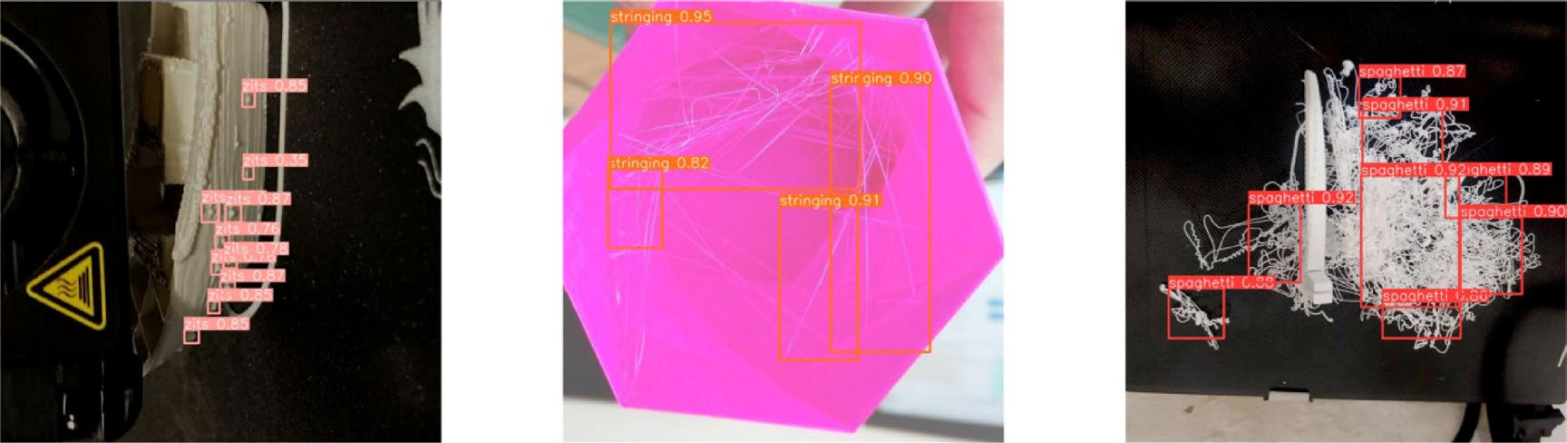
Detection visualization.

After training, the improved YOLO-AMI model was used to detect defects in additive-manufacturing products. The model achieved a precision of 87.1%, this high precision specifically counters the concern that NWD-based Gaussian modeling might cause “hallucinations” (false positives) on rough surfaces. By smoothing out high-frequency texture noise through distribution modeling, NWD prevents the model from overfitting to surface roughness, triggering detections only when significant morphological deviations occur, a recall of 83.2%, an mAP@0.5 of 85.5%, and an mAP0.5:0.95 of 53.8%. Crucially, for industrial deployment, the criticality of defects varies significantly. “Spaghetti” represents a catastrophic failure that necessitates immediate print termination to prevent equipment damage, whereas “zits” are typically cosmetic flaws. Therefore, missing a spaghetti defect (False Negative) is far more detrimental than missing zits. To validate the model’s safety reliability, we conducted a per-class analysis of the recall rates. The quantitative results indicate that the model achieves a remarkably high Recall of 94.0% for the “Spaghetti” class, corresponding to a minimal False Negative Rate of 6%. In comparison, the Recall for “zits” is 89.0%. This performance disparity demonstrates that the model is highly sensitive to critical failures, ensuring operational safety by reliably triggering stops for major anomalies, while maintaining balanced performance on cosmetic defects. Prediction results are shown in Fig. 9. Table 4 presents the detailed statistics of the annotated instances. As shown, the dataset exhibits significant challenges characteristic of industrial visual inspection: Severe Class Imbalance: The dataset is dominated by “Zits” (9,850 instances), which appear significantly more frequently than critical failures like “Spaghetti” (1,245 instances). Prevalence of Tiny Objects: Crucially, 92.5% of the “Zits” instances are classified as “small objects” (area $< 32^{2}$ px) according to the MS COCO definition. These minute targets often occupy less than 0.1% of the image area. Scale Variation: In contrast, “Stringing” defects exhibit extreme aspect ratios, often spanning large areas but with very few pixels in width. These statistical characteristics—specifically the predominance of tiny objects and class imbalance—justify the necessity of our architectural choices, such as NWD (for scale-invariant loss) and AFPN (for small feature preservation).

**Table 4:** Detailed statistics of the dataset categories and scales.

Defect category	Number of instances	Small object ratio (${<32}^{2}$ px)	Morphological characteristics
Spaghetti	1245	4.8%	Large, chaotic, spans globally
Zits	9850	92.5%	Tiny, dense, local protrusions
Stringing	3180	18.2%	Long, thin, high aspect ratio
Total	14,275	–	–

### Effect of network architectures

**Fig. 10:**
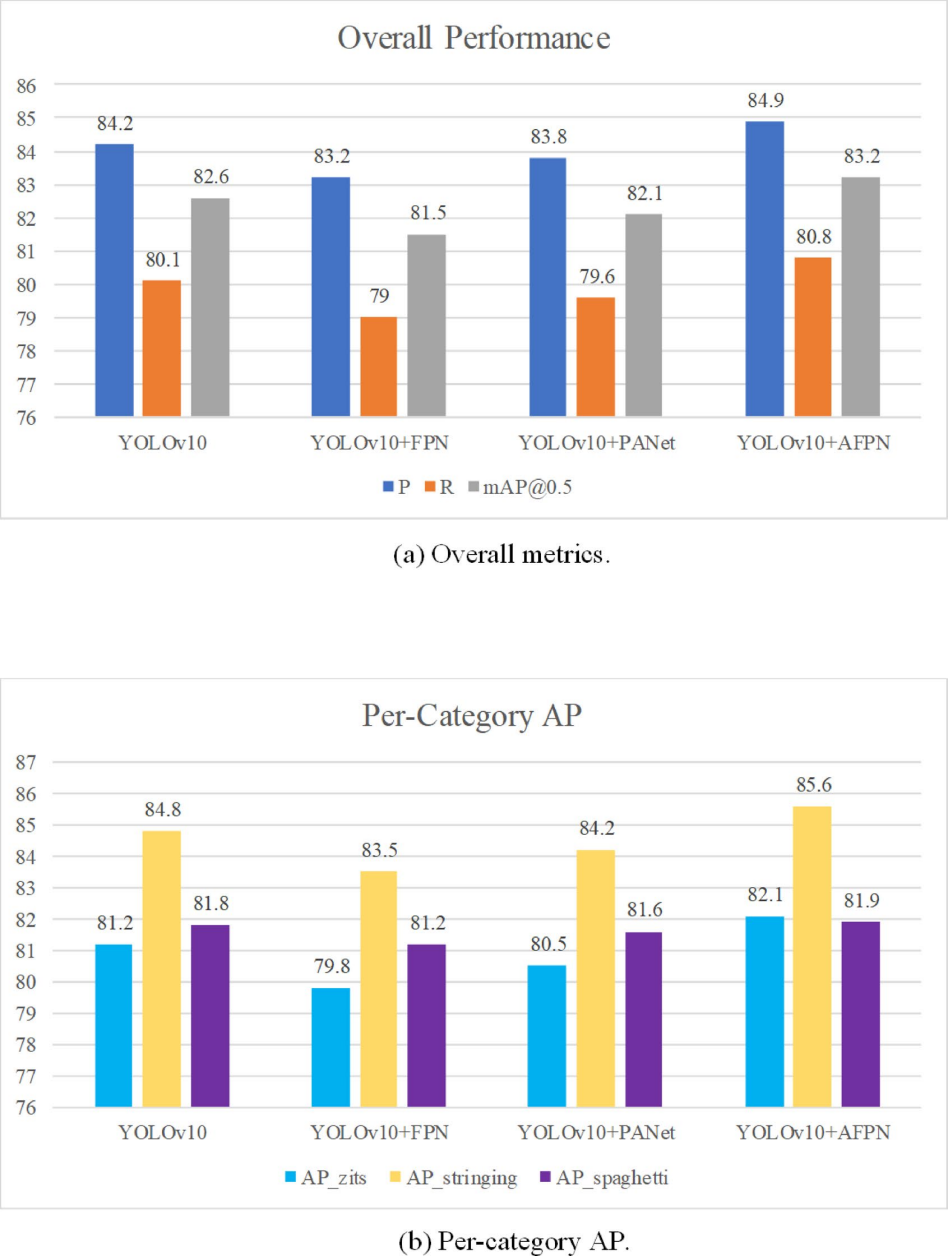
Ablation on neck structures.

To systematically evaluate the impact of different network structures on model detection performance, YOLOv10 + FPN^28^, YOLOv10 + PANet^29^, and YOLOv10 + AFPN were selected for comparison. Results are presented in Table 5 and Fig. 10.

**Table 5:** Performance comparison of different neck architectures.

Model	P/%	R/%	mAP@0.5/%	mAP@0.5:0.95/%	AP_zits/%	AP_stringing/%	AP_spaghetti/%
YOLOv10	84.2	80.1	82.6	49.5	81.2	84.8	81.8
YOLOv10 + FPN	83.2	79.0	81.5	47.9	79.8	83.5	81.2
YOLOv10 + PANet	83.8	79.6	82.1	48.7	80.5	84.2	81.6
YOLOv10 + AFPN	84.9	80.8	83.2	50.3	82.1	85.6	81.9

The results indicate that the AFPN module significantly improves detection performance. Compared with the original YOLOv10 model, the optimized neck network structure produced a 0.7% increase in both precision and recall and a 0.6% increase in mAP@0.5.

Simultaneously, superior Average Precision (AP) was achieved for specific defect classes: zits (82.1%), stringing (85.6%), and spaghetti (81.9%).

In contrast to the AFPN module, integrating FPN and PANet into YOLOv10 led to varying degrees of decline in both detection precision and recall, with no observed improvement in class-specific target detection.

AFPN demonstrated significantly superior performance compared with FPN and PANet for additive-manufacturing defect detection, thereby enhancing the capabilities of the original model; given its outstanding performance in detection accuracy and its structural complexity profile, AFPN was selected as the designated network structure for the proposed model.

### Comparison on attention mechanisms

**Fig. 11:**
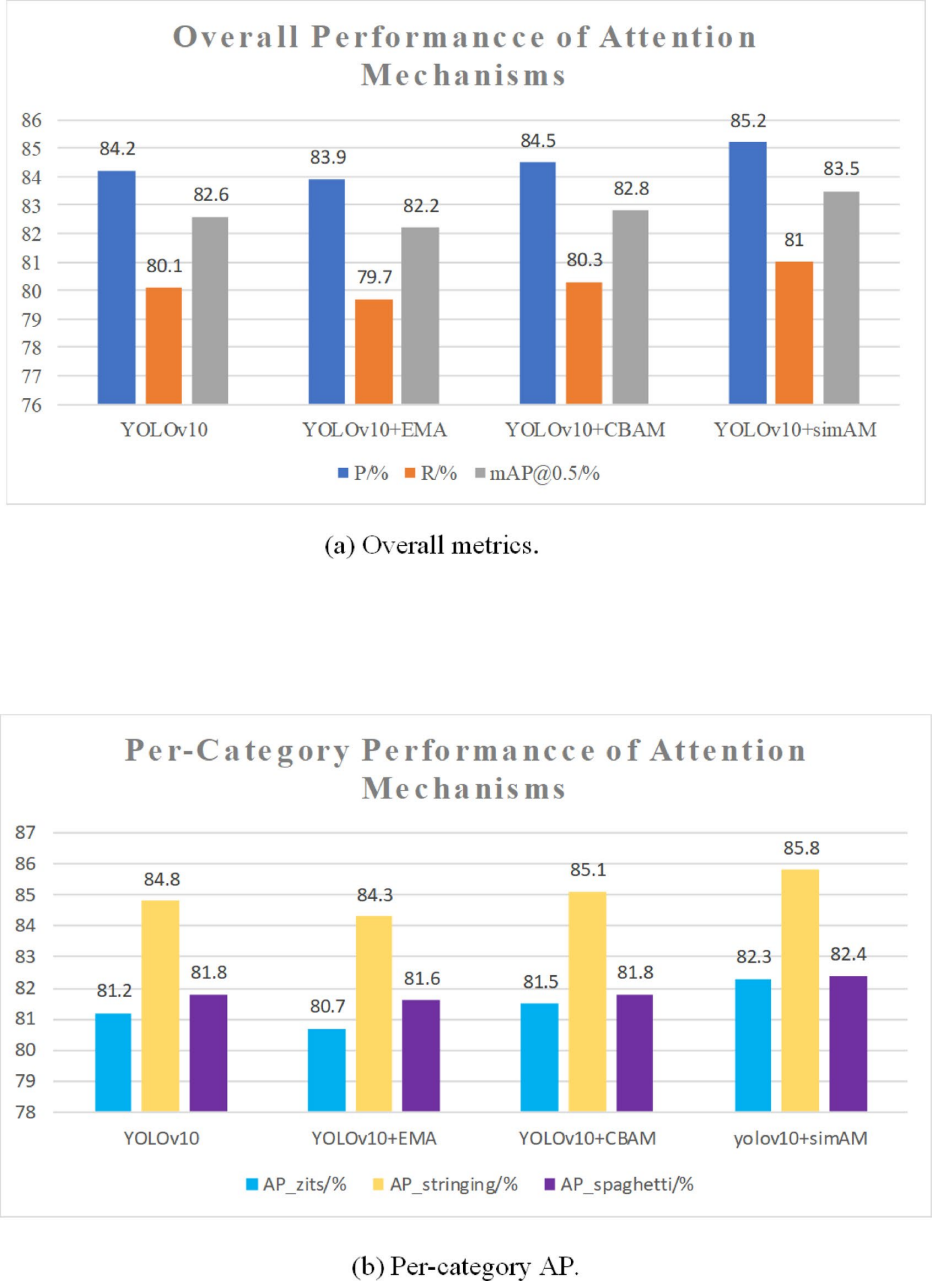
Ablation on attention mechanisms.

To further investigate the impact of different attention mechanisms on model performance, three mechanisms were compared: EMA (Exponential Moving Average)^30^, CBAM (Convolutional Block Attention Module)^31^, and SimAM (Normalized Wasserstein Distance). Results are shown in Table 6 and Fig. 11.

**Table 6:** Performance comparison of different attention mechanisms.

Model	P/%	R/%	mAP@0.5/%	mAP@0.5:0.95/%	AP_zits/%	AP_stringing/%	AP_spaghetti/%
YOLOv10	84.2	80.1	82.6	49.5	81.2	84.8	81.8
YOLOv10 + EMA	83.9	79.7	82.2	48.9	80.7	84.3	81.6
YOLOv10 + CBAM	84.5	80.3	82.8	49.8	81.5	85.1	81.8
YOLOv10 + SimAM	85.2	81.0	83.5	50.6	82.3	85.8	82.4

The results indicate that the model constructed with YOLOv10 + SimAM achieved the best performance, with a precision of 85.2% and a recall of 81.0%, corresponding to increases of 1.0% and 0.9%, respectively, compared with the original model (YOLOv10). his outcome demonstrates that adding the SimAM module to YOLOv10 improves precision and enhances the model’s ability to detect missed targets. For category-level detection, AP scores after adding SimAM were 82.3%, 85.8%, and 82.4%, representing improvements of 1.1%, 1.0%, and 0.6% over the original model.

The model with EMA added to YOLOv10 showed no improvement in detection performance—neither in precision, recall, mAP@0.5, nor in per-class AP—and was therefore not included in the final model.

After adding CBAM to YOLOv10, improvements were observed to varying degrees in precision, recall, mAP@0.5, AP_zits, and AP_stringing. However, the overall detection performance of the CBAM-enhanced model (precision, recall, mAP@0.5, and per-class AP) remained lower than that of the SimAM-enhanced model. Moreover, AP for the ‘spaghetti’ class showed no significant improvement following CBAM integration.

In summary, these experimental comparisons demonstrate that integrating SimAM into the YOLOv10 framework significantly enhances overall performance and substantially improves detection capability in complex industrial environments. Consequently, SimAM was adopted as the attention mechanism for the model.

### Analysis of different loss functions

**Fig. 12:**
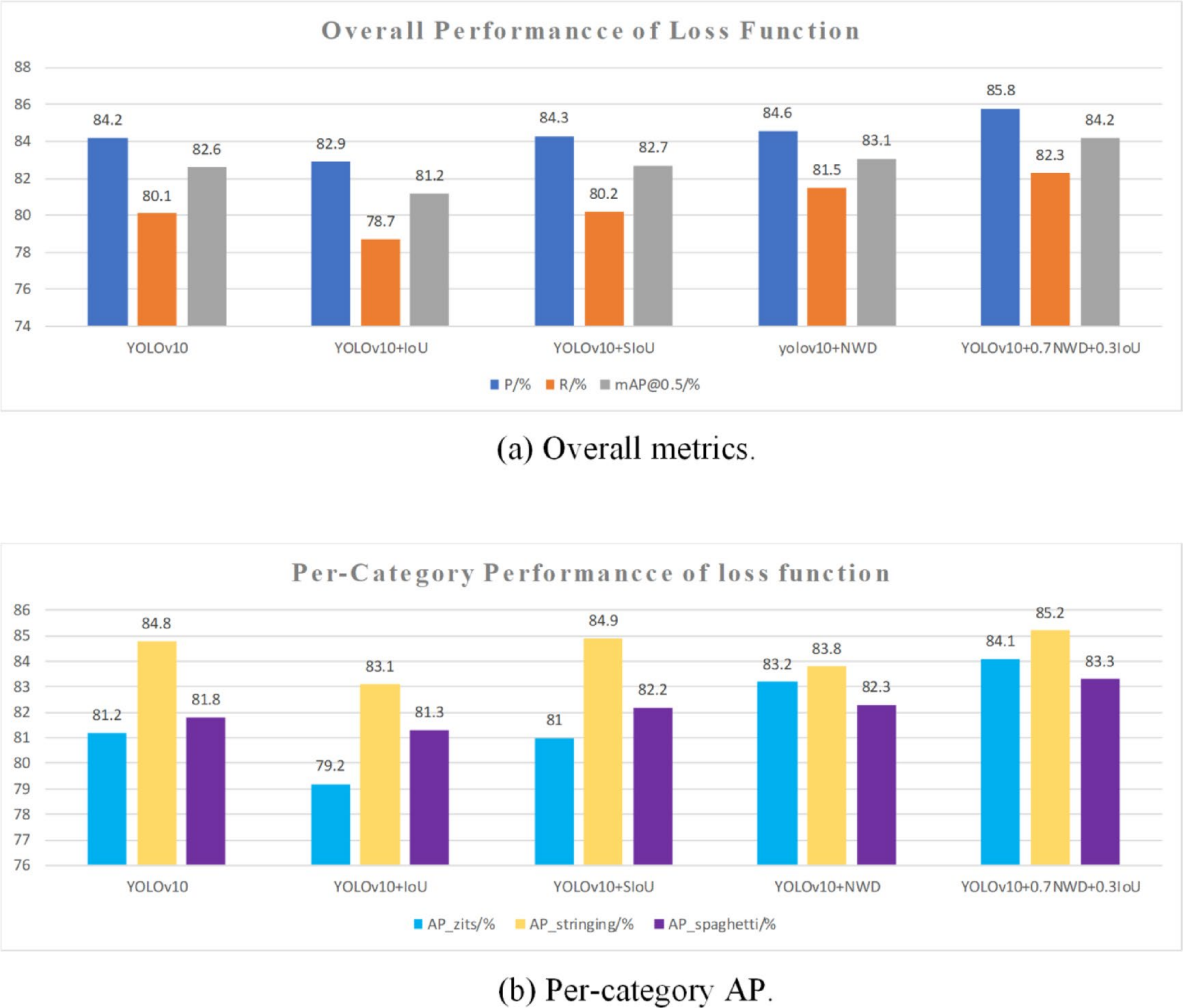
Ablation on loss functions.

The YOLOv10 model was evaluated with several loss functions—IoU^32^,SIoU^33^,NWD, and the composite 0.7NWD + 0.3IoU used in this study—and a comparative analysis was conducted; results are presented in Table 7 and Fig. 12.

**Table 7:** Performance comparison of different loss functions.

Model	P/%	R/%	mAP@0.5/%	mAP@0.5:0.95/%	AP_zits/%	AP_stringing/%	AP_spaghetti/%
YOLOv10	84.2	80.1	82.6	49.5	81.2	84.8	81.8
YOLOv10 + IoU	82.9	78.7	81.2	47.5	79.2	83.1	81.3
YOLOv10 + SIoU	84.3	80.2	82.7	49.3	81.0	84.9	82.2
YOLOv10 + NWD	84.6	81.5	83.1	50.1	83.2	83.8	82.3
YOLOv10 + 0.7NWD + 0.3IoU	85.8	82.3	84.2	51.4	84.1	85.2	83.3

Results in Table 7 show that the loss function used here (0.7NWD + 0.3IoU) provides substantial performance gains over the original YOLOv10. After incorporating 0.7NWD + 0.3IoU, the model achieved a precision of 85.6%, a recall of 82.3%, and an mAP@0.5 of 84.2%; AP_zits, AP_stringing, and AP_spaghetti reached 84.1%, 85.2%, and 83.3%, respectively. Relative to the original model, these metrics correspond to improvements of 1.6%, 2.2%, 1.6%, 2.9%, 0.4%, and 1.5%, The concurrent increase in precision and recall indicates marked improvements in detection accuracy and in the model’s ability to reduce missed detections. The improved model produced a notable increase in AP_zits together with gains in AP_stringing and AP_spaghetti. This outcome demonstrates a clear enhancement in the model’s capability to detect small-scale targets.

By contrast, adding the IoU loss alone led to declines in certain metrics, including precision and recall. When SIoU was added alone, model precision increased by 0.1% and recall increased by 0.4%. For class-specific AP, SIoU caused a 0.2% decrease for AP_zits, a 0.1% increase for AP_stringing, and a 0.4% increase for AP_spaghettiAfter adding the IoU function, the model showed varying degrees of decline in metrics such as precision and recall. When NWD was added alone, model precision increased by 0.4% and recall increased by 1.4%. For class-specific AP, NWD raised AP_zits by 2.0% and AP_spaghetti by 0.5%, while AP_stringing decreased by 1.0%. These experiments illustrate that the NWD module delivers particularly strong gains for detecting small targets such as zits.

Given the superior performance and robustness obtained with 0.7NWD + 0.3IoU, this loss configuration was adopted for the YOLO-AMI model.

### Comparison with state-of-the-art models

To comprehensively evaluate the proposed model, several mainstream state-of-the-art object detectors were included for comparison: RetinaNet-R50^34^, Faster R-CNN-R50^35^, YOLOv5^36^, YOLOv8^37^, YOLOv10, RT-DETR-L^38^, Yolov11^39^ and the proposed YOLO-AMI. Comparative metrics comprised Precision, Recall, mAP@0.5, mAP@0.5:0.95, Params, FLOPs, and FPS. To ensure a rigorously fair and standardized comparison, all models were trained and evaluated from scratch under the exact same hardware (NVIDIA RTX 3060) and software environment. During the training phase, all baseline models and our YOLO-AMI strictly shared unified hyperparameters, including an input image resolution of 640 × 640 pixels, 300 training epochs, a batch size of 8, and the SGD optimizer with identical data augmentation strategies. For the inference phase, the post-processing settings were standardized. Crucially, the FPS measurements for all models were evaluated under identical conditions (batch size of 1, FP16 precision) and explicitly included the time consumed by NMS post-processing.

**Fig. 13:**
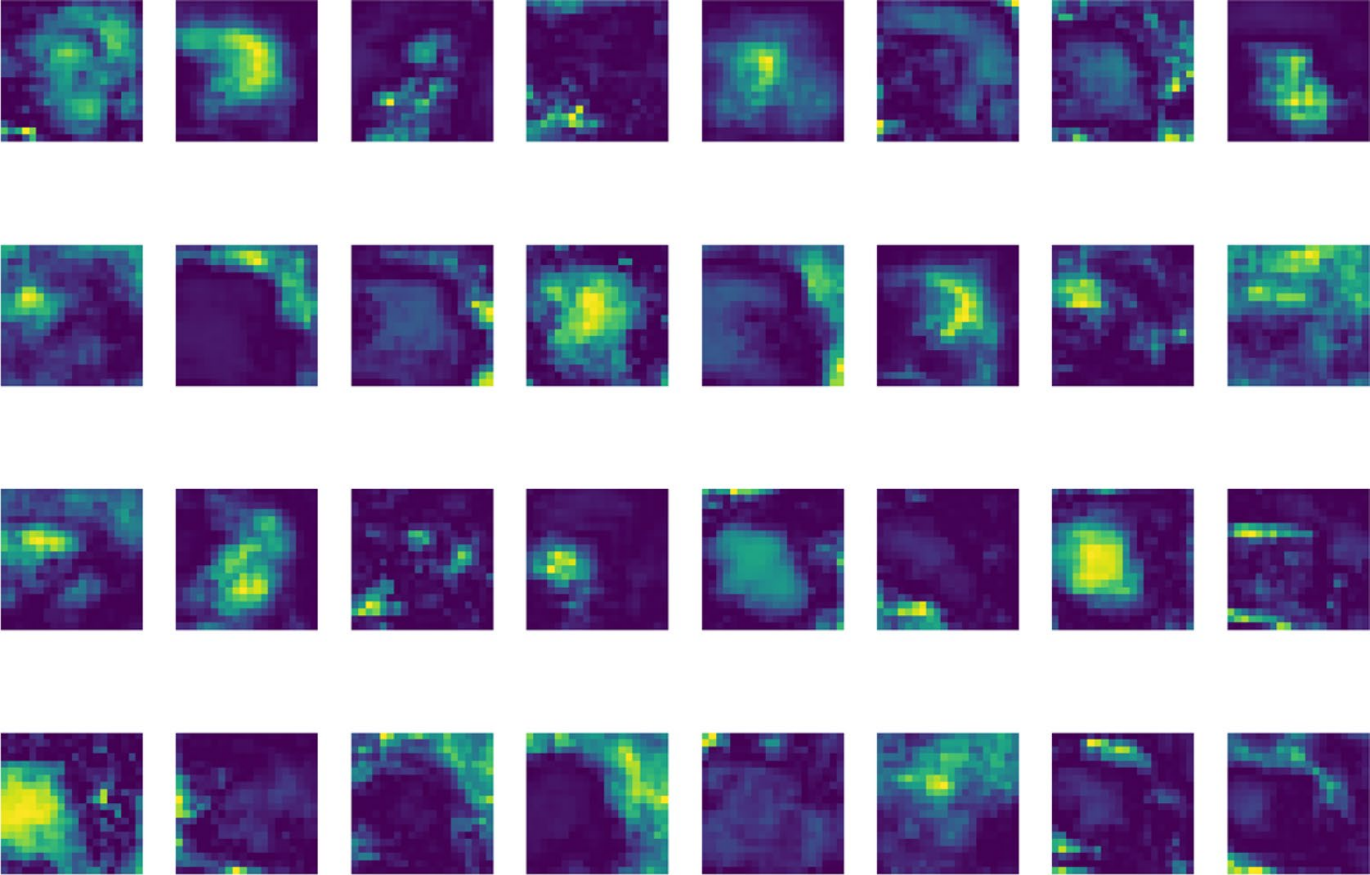
Comparison of precision, recall, and mAP@0.5 with SOTA models.

As shown in Table 5 and Fig. 13, YOLO-AMI performs strongly across these metrics, indicating high efficiency while maintaining high accuracy.

Experimental results show that YOLO-AMI significantly outperforms other compared models on key detection metrics, including Precision, Recall, mAP@0.5, and the more comprehensive mAP@0.5:0.95.

Notably, YOLO-AMI achieves outstanding computational efficiency without sacrificing detection accuracy. With only 8.6 M parameters and a computational cost of 78.5G FLOPs, YOLO-AMI represents a well-balanced trade-off between accuracy and efficiency and offers strong deployment potential.

**Fig. 14:**
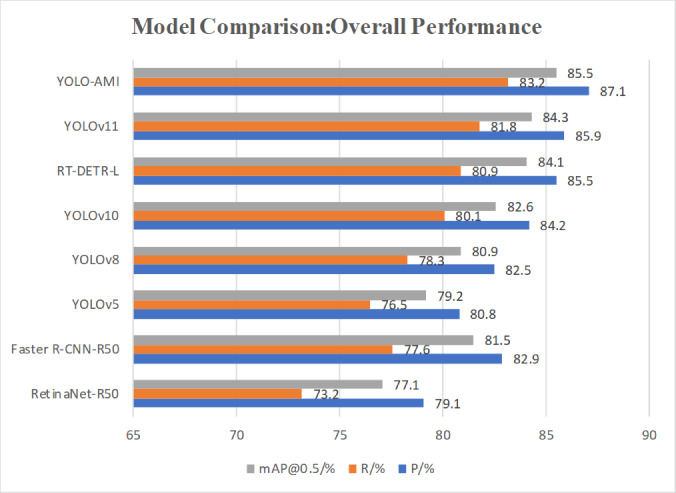
Visualization of deep feature activation maps showing the model’s focus on defects while suppressing background texture.

As indicated in Table 8, YOLO-AMI attains an mAP@0.5 of 85.5%, which is 1.4% and 1.2% higher than RT-DETR-L (84.1%) and YOLOv11 (84.3%), respectively. Although RT-DETR-L is a powerful contender with global context modeling, YOLO-AMI achieves a higher mAP@0.5 (85.5% vs. 84.1%). This outcome can be attributed to two primary factors: First, the inductive bias of the CNN architecture allows for more efficient feature learning on the limited training data compared to the data-hungry Transformer architecture. To empirically validate this hypothesis and verify that the proposed model is focusing on the correct defect features rather than background noise, we visualized the deep-layer feature activation maps (see Fig. 14.Visualization of deep feature activation maps showing the model’s focus on defects while suppressing background texture.). As observed in the visualization, the specific defect regions (e.g., zits and stringing) exhibit high activation values (highlighted regions), while the periodic layer striations of the print bed remain suppressed (dark regions). This confirms that YOLO-AMI, leveraging the inductive bias of CNNs and the spatial suppression capability of SimAM, effectively filters out texture noise and concentrates on the defect morphology. Second, our specific enhancements (NWD and SimAM) effectively target the small and irregular defects that generic models may overlook.

**Table 8:** Comparison with state-of-the-art detectors on the AM dataset.

Model	P/%	R/%	mAP@0.5/%	mAP@0.5:0.95/%	Params/M	FLOPs/G	FPS
RetinaNet-R50	79.1	73.2	77.1	42.5	38.2	155.3	40.8
Faster R-CNN-R50	82.9	77.6	81.5	48.0	41.5	182.4	20.3
YOLOv5	80.8	76.5	79.2	44.8	7.5	16.5	120.4
YOLOv8	82.5	78.3	80.9	47.2	11.1	28.6	100.5
YOLOv10	84.2	80.1	82.6	49.5	8.2	8.2	109.3
RT-DETR-L	85.5	80.9	84.1	51.9	33.0	110.7	74.4
YOLOv11	85.9	81.8	84.3	52.1	25.5	78.5	79.6
YOLO-AMI	87.1	83.2	85.5	53.8	8.6	8.4	105.6

YOLO-AMI also leads in recall. The recall for YOLO-AMI is 83.2%, which is 1.4% and 2.3%higher than those of YOLOv11 and RT-DETR-L, respectively.

**Fig. 15:**
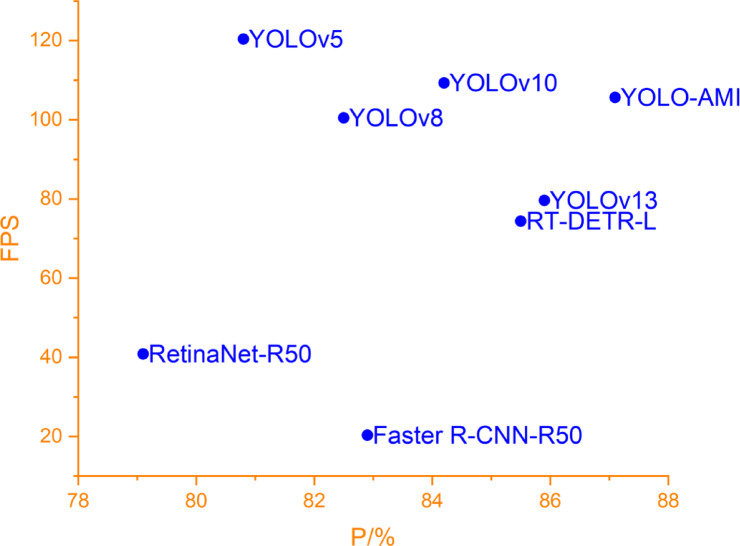
Precision vs. speed (FPS) trade-off.

In terms of inference speed, YOLO-AMI reaches 105.6 FPS. Although slightly slower than YOLOv5 and YOLOv10 (see Fig. 15.), the substantial accuracy gains—combined with very low memory usage and fast inference—support YOLO-AMI’s suitability for deployment on resource-constrained devices.

In summary, comparisons with mainstream state-of-the-art models confirm that YOLO-AMI achieves top-tier detection accuracy (mAP@0.5 = 85.5%) and recall, while also offering advantages in model complexity (8.6 M parameters) and inference speed (105.6 FPS), thus achieving an optimal balance between precision and efficiency.

### Deployment scenarios and economic analysis

Deployment Scenarios and Economic Analysis We acknowledge that deploying a dedicated high-end GPU (like the RTX 3060) for a single 3D printer would be economically inefficient due to hardware costs and power consumption. Following the industrial trend toward centralized management, we frame our proposed YOLO-AMI within the context of “High-End Industrial Workstations” for monitoring large-scale 3D print farms, rather than as a standalone edge solution.

In this centralized architecture, the high inference speed of YOLO-AMI (105.6 FPS) becomes a critical advantage. Since the FDM printing process has relatively slow dynamics, a sampling rate of 1 FPS is typically sufficient for defect detection. This implies that a single RTX 3060 workstation can effectively process video streams from over 100 printers simultaneously. By amortizing the hardware and energy costs across a large fleet of printers, the cost per monitored unit becomes negligible. Furthermore, while this study validates the model on a workstation, the model’s lightweight nature (8.6 M parameters) retains the potential for future migration to resource-constrained edge devices if decentralized deployment is required.

While this study focuses on the accuracy and speed of the detection algorithm on a centralized workstation, the system’s theoretical latency supports its viability for future closed-loop control. The inference time of YOLO-AMI is approximately 9.5 ms (105.6 FPS). Assuming a typical network transmission and control command latency of ~ 200 ms in a local area network (LAN), the total system response time would remain under 0.5 s.

Unlike high-speed CNC machining where milliseconds matter to prevent catastrophic collisions, FDM 3D printing is a relatively low-dynamic process (typical print speeds of 50–60 mm/s). Defects like “spaghetti” accumulate gradually over minutes. Therefore, a sub-second response time is more than sufficient to trigger an emergency stop before the defect accumulation poses a risk of nozzle collision or severe equipment damage. The current system provides real-time alerts, and fully integrated closed-loop control will be implemented in future work.

Furthermore, we analyzed the trade-off between Precision and Recall specifically in the context of waste mitigation. In additive manufacturing, the cost of a False Negative (missing a spaghetti defect) is substantial, resulting in wasted filament, energy, and machine time. Conversely, the cost of a False Positive is relatively low, incurring only a brief pause for manual verification. Therefore, prioritizing Recall is essential for effective waste mitigation. Our model’s high Recall of 94.0% for critical defects ensures that material-wasting failures are intercepted early. Simultaneously, the high Precision of 87.1% ensures that this sensitivity does not result in excessive operational downtime due to false alarms, achieving an optimal economic balance.

### Failure case analysis and limitations

While YOLO-AMI demonstrates robust overall performance, a systematic analysis of boundary cases reveals specific scenarios that still lead to missed detections (False Negatives, FN) or false alarms (False Positives, FP). Understanding these failure modes is crucial for future system refinement.

Missed detections predominantly occur under two extreme conditions. First, transient physical occlusion remains a challenge. If a micro-defect (e.g., a small zit) forms directly behind the moving extruder nozzle relative to the camera’s line of sight, the single-view setup cannot capture it until the print head moves away. Second, extreme low-contrast environments—such as printing with black filament on a dark, unlit print bed—can cause early-stage stringing or tiny zits to blend into the background, falling below the detectable gradient threshold of the network.

False alarms, though rare (as evidenced by the 87.1% Precision), are typically triggered by background texture mimicry. Residue from bed adhesives (e.g., glue stick marks or Kapton tape bubbles) or the inherent granular texture of PEI build plates can occasionally be misclassified as “zits” by the network, especially during the first layer deposition. Furthermore, legitimate but excessively thin filament trails produced during normal extruder travel moves can sometimes cross the semantic boundary and be falsely flagged as “stringing.”

These failure cases highlight the inherent limitations of monocular spatial detection. As noted in our future work, mitigating these specific errors will require integrating temporal tracking (to distinguish static bed residue from newly formed dynamic defects) and multi-view camera arrays (to eliminate occlusion blind spots).

## Conclusion

This study addresses product-quality challenges in additive manufacturing by proposing an automated, high-precision, deep-learning-based defect-detection method, thereby advancing intelligence and automation in the AM domain. A dataset comprising three representative AM defect types was constructed, and the YOLOv10 architecture was optimized by integrating an AFPN module, the SimAM attention mechanism, and an improved composite loss function, resulting in the YOLO-AMI model tailored for industrial inspection. Ablation studies demonstrate that each modification significantly improves model performance.

Comparative experiments show that YOLO-AMI attains state-of-the-art results on key metrics — Precision, Recall, mAP@0.5, and mAP@0.5:0.95— outperforming mainstream detectors. Regarding efficiency, YOLO-AMI combines a compact parameter footprint (8.6 million parameters) with a high inference rate (105.6 FPS), while substantially improving detection accuracy, thus balancing speed and precision for industrial applications.

Future work includes: (I) dataset expansion to incorporate a broader range of defect types and improve generalization; (II) multimodal fusion with modalities such as infrared thermography or acoustic sensing to enable earlier and more accurate defect detection; (III) field deployment on 3D-printer controllers or edge devices to provide real-time feedback and closed-loop control; and (IV) addressing the transient occlusion limitations of the current monocular setup by implementing a multi-camera array for multi-view redundancy. In conclusion, the YOLO-AMI model constitutes an effective, efficient, and reliable technical solution for additive manufacturing, with practical significance for advancing smart manufacturing and Industry 4.0.

## Supplementary Information

Below is the link to the electronic supplementary material.

Supplementary Material 1

## Data Availability

The data and code presented in this study are available within the article and its supplementary material.
